# Comparative safety of concurrent adjuvant trastuzumab emtansine vs trastuzumab with radiotherapy in HER2-positive early breast cancer

**DOI:** 10.1093/oncolo/oyag372

**Published:** 2026-09-21

**Authors:** Mine Aysan, Bahadir Koylu, Ayberk Bayramgil, Dilek Unal, Banu Karaalioğlu, Ahmet Bilici, Fatih Selçukbiricik, Ozcan Yildiz, Ömer Fatih Olmez

**Affiliations:** Department of Medical Oncology, Faculty of Medicine, Medipol University, Istanbul, 34083, Türkiye; Department of Medical Oncology, Faculty of Medicine, Koc University, Istanbul, 34460, Türkiye; Department of Medical Oncology, Umraniye Training and Research Hospital, Istanbul, Türkiye; Department of Radiation Oncology, Faculty of Medicine, Medipol University, Istanbul, 34083, Türkiye; Department of Radiology, Faculty of Medicine, Medipol University, Istanbul, 34083, Türkiye; Department of Medical Oncology, Faculty of Medicine, Medipol University, Istanbul, 34083, Türkiye; Department of Medical Oncology, Faculty of Medicine, Koc University, Istanbul, 34460, Türkiye; Department of Medical Oncology, Faculty of Medicine, Medipol University, Istanbul, 34083, Türkiye; Department of Medical Oncology, Acibadem Maslak Hospital, Istanbul, 34398, Türkiye

**Keywords:** epidermal growth factor receptor 2, early breast cancer, adjuvant therapy, radiotherapy, trastuzumab emtansine, toxicity, safety

## Abstract

**Background:**

Real-world comparative data on the safety of adjuvant trastuzumab emtansine (T-DM1) vs trastuzumab with concurrent radiotherapy (RT) in HER2-positive early breast cancer (BC) remain limited.

**Methods:**

In this retrospective, multicenter study, we included 133 patients with HER2-positive early-stage BC treated with neoadjuvant therapy, surgery, and adjuvant systemic therapy administered concurrently with RT at 4 hospitals. Patients received adjuvant T-DM1 (*n* = 50) if residual disease was present, or trastuzumab (*n* = 83) if a pathologic complete response was achieved. Toxicities were graded according to CTCAE criteria. A *post-hoc* power analysis indicated 80% power to detect an absolute between-group difference in toxicity rates of ≥ 25%.

**Results:**

The T-DM1 group had poorer performance status, higher nodal stage, and more intensive neoadjuvant regimens. RT parameters were balanced between groups (median dose 50 Gy, 25 fractions; *P* > .05 for all comparisons). T-DM1 was associated with higher rates of thrombocytopenia (70% vs 36.1%, *P* < .001), ALT elevation (20% vs 4.8%, *P* = .008), AST elevation (24.5% vs 2.4%, *P* < .001), and early pulmonary toxicity (48.8% vs 24.1%, *P* = .017), with events predominantly grade 1. In sensitivity analyses adjusting for pertuzumab exposure, T-DM1 associations remained significant for thrombocytopenia (adjusted odds ratio [OR] = 3.85, *P* = .001), ALT elevation (adjusted OR = 4.12, *P* = .026), and early pulmonary toxicity (adjusted OR = 2.64, *P* = .034).

**Conclusion:**

Concurrent adjuvant T-DM1 and RT demonstrated higher but manageable hematologic, hepatic, and pulmonary toxicity compared with trastuzumab. These hypothesis-generating findings require validation in prospective studies.

## Graphical abstract

**Graphical Abstract:**
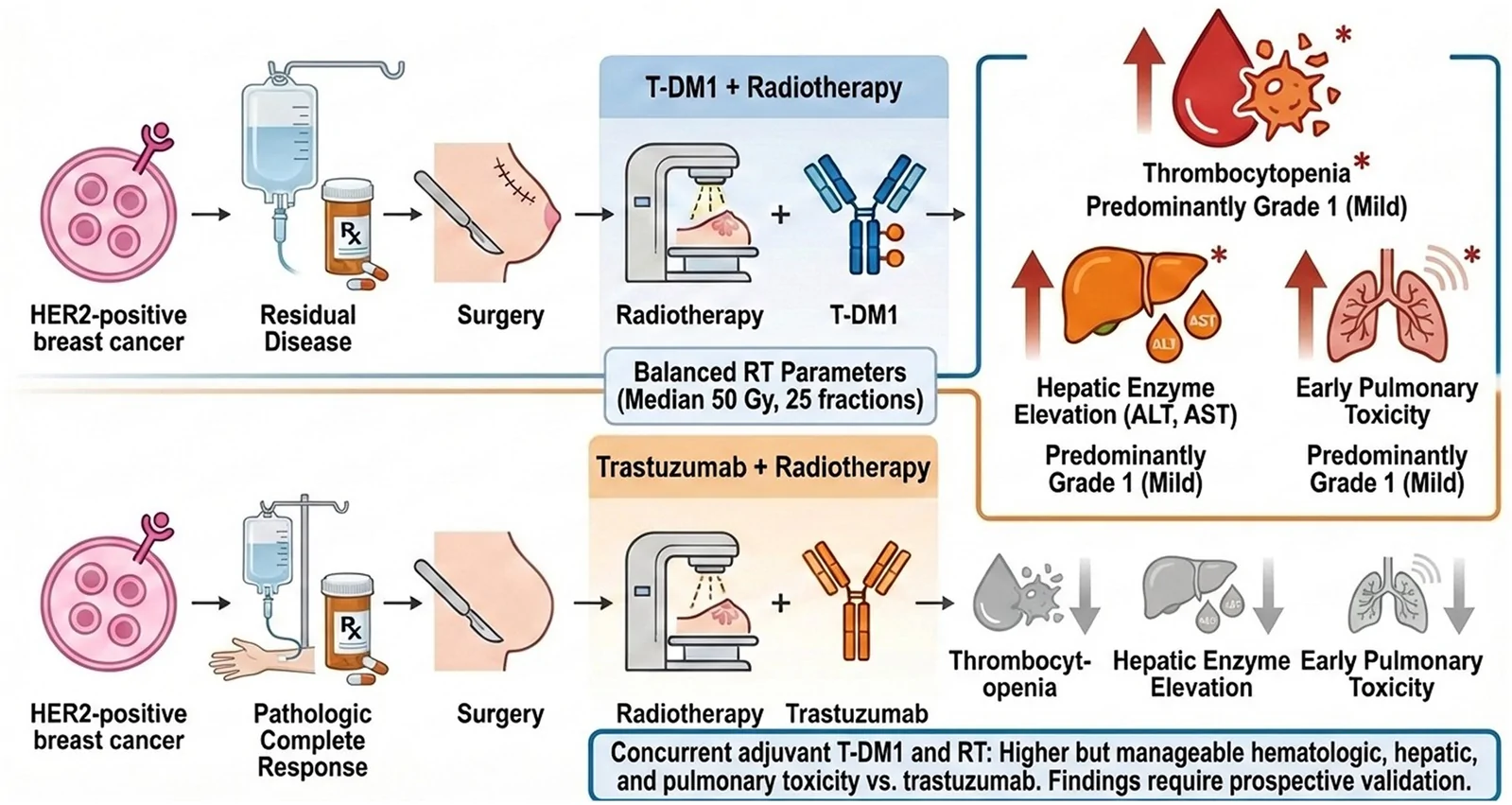

Implications for practice Concurrent administration of adjuvant trastuzumab emtansine (T-DM1) and radiotherapy in HER2-positive early-stage breast cancer is feasible and generally well tolerated, though associated with higher rates of thrombocytopenia, hepatic enzyme elevation, and mild pulmonary toxicity compared with trastuzumab. These findings emphasize the importance of vigilant hematologic, hepatic, and pulmonary monitoring during concurrent therapy, particularly in patients with reduced performance status, baseline cytopenias, or organ dysfunction. Optimal monitoring strategies (frequency, specific assessments, and intervention thresholds) require investigation through prospective research to optimize the safe integration of T-DM1 with postoperative radiotherapy in routine clinical practice.

## Introduction

Human epidermal growth factor receptor 2 (HER2) overexpression and/or *ERBB2* gene amplification occurs in approximately 15%-20% of breast cancer (BC) cases and confers an aggressive phenotype.^1^^,^^2^ While HER2-targeted therapies, particularly trastuzumab-based regimens, have dramatically improved outcomes and represent current standard of care,^3^^,^^4^ approximately 40-50% of patients with HER2-positive early-stage BC have residual disease after neoadjuvant therapy and surgery.^5^ Notably, in randomized trials of neoadjuvant chemotherapy combined with HER2-directed therapy, patients with residual invasive disease after treatment had a 3-year disease-free survival (DFS) of approximately 80%, compared with ∼90% among those achieving a pathological complete response.^6^ The landmark KATHERINE trial established trastuzumab emtansine (T-DM1) as the preferred adjuvant therapy for patients with HER2-positive early BC who have residual invasive disease after neoadjuvant chemotherapy and HER2-targeted therapy.^7^ T-DM1 is an antibody-drug conjugate that combines trastuzumab (targeting HER2-positive tumor cells) with DM1 (emtansine), a potent maytansinoid that inhibits microtubule polymerization, leading to cell cycle arrest and apoptosis.^8^ In the KATHERINE trial, T-DM1 reduced the risk of invasive disease-free survival events by 50% compared with trastuzumab alone (3-year DFS: 88.3% vs 77.0%, *P* < .001), leading to its incorporation into international treatment guidelines.^7^ In contrast, patients achieving pCR may continue to receive standard trastuzumab-based adjuvant therapy.^9^

Radiotherapy (RT) remains a cornerstone of BC treatment and is typically administered concurrently with adjuvant systemic therapy to maximize local control.^10^ While concurrent trastuzumab with RT is considered safe and effective,^11^^,^^12^ limited data exist for T-DM1 administered concurrently with RT. T-DM1’s unique mechanism-delivering the potent microtubule inhibitor DM1 directly to HER2-positive cells-raises theoretical concerns about enhanced radiosensitivity and toxicity.^13^^,^^14^ Although a small retrospective series and a recent meta-analysis suggest feasibility with low rates of severe toxicity (grade ≥ 3 rates of ∼7%^15^ and grade 3+ skin toxicity 1%, pneumonitis <1%^16^), direct toxicity comparisons with standard trastuzumab-based regimens during concurrent RT are lacking. As adjuvant T-DM1 becomes increasingly adopted for patients with residual disease following neoadjuvant treatment^17^ real-world safety data are essential for optimal monitoring protocols. In this study, we directly compared the toxicity profiles of concurrent adjuvant T-DM1 plus RT vs concurrent adjuvant trastuzumab plus RT in patients with HER2-positive early-stage breast cancer who had received neoadjuvant therapy followed by surgery, and identified predictors of toxicity to inform future prospective trials.

## Methods

### Study design and setting

This real-world, retrospective, multicenter study was conducted at Medipol University, Koç University, Ümraniye Training and Research Hospital, and Acıbadem Maslak Hospital (Istanbul and Türkiye). The protocol was approved by the Medipol University Faculty of Medicine Institutional Review Board (No. E-10840098-202.3.02-4365). Informed consent was waived due to the retrospective design.

### Patient selection

The study participants were identified via retrospective review of clinical records collected between 2010 and 2024 from patients with HER2-positive early-stage BC who received neoadjuvant chemotherapy, underwent definitive surgery, and subsequently received adjuvant therapy including RT. The study period (2010-2024) spans both the years preceding and following the publication of the KATHERINE trial in 2019.^7^ Before 2019, T-DM1 was occasionally prescribed off-guideline, informed by emerging evidence from the EMILIA trial in advanced BC.^18^ From 2019 onward, T-DM1 was adopted as the standard of care for patients with residual invasive disease.^7^ Treating oncologists made therapy decisions on the basis of individual patient characteristics, pathologic response, and institutional protocols. Inclusion criteria were: (1) histologically confirmed invasive BC; (2) HER2-positive status defined by immunohistochemistry 3+ or amplification by *in situ* hybridization; (3) clinical stage I-III disease at diagnosis; (4) completion of neoadjuvant chemotherapy with HER2-targeted therapy; (5) definitive surgical treatment (mastectomy or lumpectomy); (6) receipt of adjuvant HER2-targeted therapy with concurrent RT; and (7) complete 1-year follow-up data for toxicity assessment. Exclusion criteria comprised: (1) metastatic disease at diagnosis; (2) prior history of breast or chest wall RT; (3) treatment discontinuation due to non-medical reasons before completing the evaluation period; (4) concurrent participation in investigational trials that might confound toxicity assessment; and (5) incomplete treatment records. A treatment record was considered incomplete if it lacked: (1) pathologic response assessment, (2) neoadjuvant chemotherapy regimen details, (3) RT dosing and field specifications, (4) baseline laboratory values (complete blood count and liver function tests), or (5) follow-up laboratory or imaging data during the toxicity assessment period. Patients enrolled in investigational trials were excluded if they received: (1) experimental agents not approved for standard care, (2) novel radiotherapy techniques under investigation, (3) investigational HER2-targeted agents beyond trastuzumab, pertuzumab, or T-DM1, or (4) any intervention that could confound standard toxicity assessment.

### Treatment protocols

Patients received neoadjuvant regimens consisting of anthracycline-based chemotherapy (doxorubicin or epirubicin), taxane-based therapy, and carboplatin as indicated. HER2-targeted therapy was administered according to standard protocols and institutional guidelines, utilizing either trastuzumab monotherapy or dual blockade with trastuzumab plus pertuzumab. Neoadjuvant pertuzumab utilization varied according to regional availability and reimbursement policies, with routine incorporation into treatment regimens commencing approximately in 2015. In patients not receiving T-DM1, pertuzumab was continued into the adjuvant phase, typically in combination with trastuzumab. Patients with residual disease received adjuvant T-DM1 monotherapy per the KATHERINE protocol.^7^ Definitive surgical treatment was performed 3-6 weeks after completion of neoadjuvant therapy and consisted of either mastectomy or breast-conserving surgery (lumpectomy) with axillary lymph node assessment.

### Adjuvant treatment assignment

Patients were stratified based on pathological response to neoadjuvant therapy. Those with residual invasive HER2-positive disease in the breast and/or axilla following neoadjuvant therapy plus trastuzumab and definitive surgery received adjuvant T-DM1 (3.6 mg/kg intravenously every 3 weeks for 14 cycles). Patients who achieved pCR, or who otherwise did not meet criteria for adjuvant T-DM1, were treated with adjuvant trastuzumab (8 mg/kg loading dose followed by 6 mg/kg intravenously every 3 weeks) to complete 1 year of HER2-targeted therapy. Participants received postoperative RT via 3D-CRT or IMRT using standard fractionation (1.8-2.0 Gy daily). Target volumes, defined by institutional guidelines, included the whole breast (post-lumpectomy) or chest wall and regional lymph nodes (post-mastectomy). Total doses ranged from 40 to 50 Gy, with 10-16 Gy tumor bed boosts delivered in breast-conserving cases when indicated.

### Data collection

Patient data were extracted from electronic medical records. Demographic and clinical characteristics included age, menopausal status, smoking history (available for 66 patients), and documented comorbidities (diabetes mellitus, coronary artery disease/hypertension, hyperlipidemia, and hypothyroidism). Disease characteristics included tumor stage (pT), nodal stage (pN), estrogen receptor (ER) and progesterone receptor (PR) status, and histologic grade (available for 121 patients). Treatment-related parameters included specific neoadjuvant chemotherapy regimens (anthracycline-based, taxane-based, and carboplatin-based), HER2-targeted therapy approaches (trastuzumab alone vs trastuzumab plus pertuzumab), surgical procedures performed (mastectomy vs lumpectomy), adjuvant therapy protocols, RT parameters, and pathologic response assessments using the Miller-Payne classification system^19^ (available for 122 patients). Patient performance status was evaluated using the Eastern Cooperative Oncology Group (ECOG) scale.^20^

### Toxicity endpoints

The primary endpoint was treatment-related toxicity rates in patients receiving adjuvant T-DM1 vs trastuzumab. Secondary endpoints included assessment of toxicity severity grades and identification of risk factors associated with treatment-related adverse events.

### Toxicity evaluation

Toxicity assessment was conducted during the 1-year period of active HER2-targeted therapy to capture acute and subacute treatment-related adverse events. Adverse events in the adjuvant T-DM1 and trastuzumab groups during 1 year of HER2-targeted therapy were assessed and graded according to the Common Terminology Criteria for Adverse Events (CTCAE), v5.0.^21^ For statistical analysis, only the first occurrence of each specific adverse event (regardless of grade) within the 1-year follow-up period was recorded and analyzed. For hematologic toxicity, complete blood counts were obtained at baseline, weekly during the first cycle, and before each subsequent cycle to assess and grade neutropenia, leukopenia, thrombocytopenia, and anemia. Hepatic toxicity was monitored through liver function tests (alanine aminotransferase [ALT] and aspartate aminotransferase [AST]) at baseline and prior to each treatment cycle. Pulmonary toxicity was assessed via systematic chest CT review at baseline, early (0-6 months), and late (6-12 months) phases. Treatment-induced lung alterations-including ground-glass opacities, consolidation, and parenchymal abnormalities within the radiation field-were graded per CTCAE v5.0 criteria^21^ with correlation to clinical symptoms. Early pulmonary toxicity was defined as new or progressive CT abnormalities observed from the start of RT to 6 months after its completion, whereas late toxicity referred to changes occurring 6-12 months post-RT. In addition, dermatologic reactions within the radiation field were assessed weekly during RT and at each follow-up visit. Finally, peripheral neuropathy was assessed through clinical examination at baseline and before each treatment cycle using standardized neurological assessments.

### Statistical analysis

Continuous variables were expressed as medians with ranges or means with standard deviations, as appropriate. Categorical variables were reported as frequencies and percentages. Between-group comparisons were performed using chi-square tests for categorical variables, and Student’s *t*-test or Mann-Whitney *U* test for continuous variables, depending on data distribution. To identify associations between adjuvant T-DM1 and observed toxicities, we performed univariable and multivariable logistic regression analysis. Multivariable logistic regression analyses were performed to assess whether treatment type remained associated with toxicity after accounting for baseline differences. Specific covariates included ECOG performance status (0 vs 1), pathological nodal stage (pN0 vs pN1-3), surgical approach (mastectomy vs lumpectomy), receipt of carboplatin (yes vs no), and dual HER2 blockade (trastuzumab alone vs trastuzumab plus pertuzumab). Results are presented as odds ratios (ORs) with 95% confidence intervals (CIs). Because use of pertuzumab differed significantly between groups, we performed sensitivity analyses adjusted for its exposure. *Post-hoc* power calculations were performed using 2-proportion tests. With our sample sizes (T-DM1, *n* = 50; trastuzumab, *n* = 83) and 2-sided significance level of 0.05, we had approximately 80% power to detect an absolute difference of 25 percentage points in toxicity rates (assuming a baseline rate of 30% in the trastuzumab group). For detecting smaller differences (15 percentage points), power was approximately 45%. For rare toxicities occurring in < 10% of patients, the study was substantially underpowered (power <30%). All calculations were performed using SPSS, version 20.0 (IBM Corp., Armonk, NY, USA). Statistical significance was defined as a *P* value < 0.05 (2-tailed).

## Results

### Patient characteristics and treatment details

Figure 1 presents the study flow diagram. A total of 133 women (mean age, 51.7 ± 12.0 years; range, 27-83 years) with HER2-positive early-stage BC were included in the study. Among the 133 included patients, smoking history was documented for 66 patients (49.6%), histologic grade for 121 patients (91.0%), and Miller-Payne classification for 122 patients (91.7%). A total of 50 patients (37.6%) were treated with concurrent adjuvant T-DM1 and RT, whereas the remaining 83 (62.4%) received concurrent adjuvant trastuzumab and RT. In the entire study cohort, the median RT dose was 1 Gy (range, 1-3 Gy), delivered over a median of 25 fractions (range, 5-32 fractions). The median planning target volume (PTV) was 50 cm^3^, while the mean RT duration was 1.14 ± 0.30 months. RT parameters were well-balanced between treatment groups. The median total RT dose was 50 Gy (range, 40-60 Gy) in both groups (*P* = .845). Median number of fractions was 25 in both groups (T-DM1: range, 20-30; trastuzumab: range, 20-33; *P* = .691). Use of IMRT vs 3D-CRT was similar (T-DM1: 42%; trastuzumab: 38%; *P* = .687). However, significant imbalances in patient, disease, and treatment characteristics were observed (Table 1). Specifically, patients in the T-DM1 group had significantly worse ECOG performance status (14.6% vs 2.4% with ECOG 1, *P* = .013), higher rates of nodal involvement with significantly different pathological N stage distribution (*P* = .006), and were more likely to undergo breast-conserving surgery (22.0% vs 3.7%, *P* = .002). As expected, the T-DM1 group demonstrated significantly different pathological responses according to Miller-Payne classification (*P* < .001), with fewer pCR (8.3% vs 67.6%) and more partial responses (43.8% vs 9.5%) compared to the trastuzumab group. In addition, the T-DM1 group received more intensive neoadjuvant chemotherapy regimens, with higher rates of carboplatin incorporation (26.0% vs 2.4%, *P* < .001) and dual HER2 blockade with trastuzumab plus pertuzumab (84.0% vs 51.8%, *P* < .001), reflecting the more aggressive nature of their disease.

**Figure 1.**
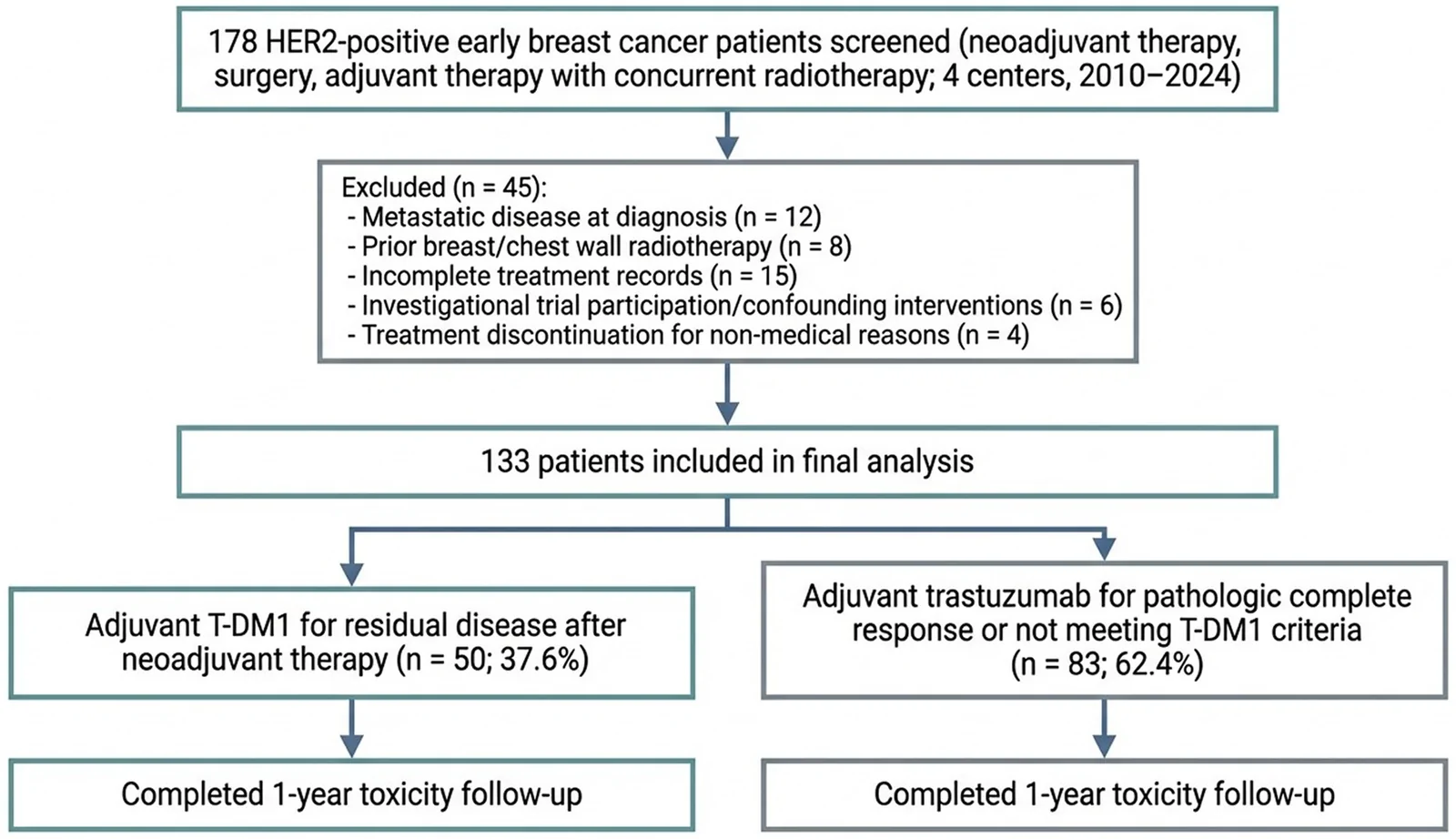
Flow of patients through the study.

**Table 1.** Patient and tumor characteristics by treatment group.

Characteristic	Entire cohort (*n* = 133)	T-DM1 (*n* = 50)	Trastuzumab (*n* = 83)	*P* value
**Menopausal status**				0.200
** Premenopausal**	81 (61.4%)	27 (54.0%)	54 (65.9%)	
** Postmenopausal**	51 (38.6%)	23 (46.0%)	28 (34.1%)	
**ECOG performance status**				**0.013**
** ECOG 0**	121 (93.1%)	41 (85.4%)	80 (97.6%)	
** ECOG 1**	9 (6.9%)	7 (14.6%)	2 (2.4%)	
**Comorbidities**				
** Diabetes mellitus**	16 (12.0%)	6 (12.0%)	10 (12.0%)	1.000
** CAD/hypertension**	31 (23.3%)	12 (24.0%)	19 (22.9%)	1.000
** Hyperlipidemia**	6 (4.5%)	4 (8.0%)	2 (2.4%)	0.197
** Hypothyroidism**	17 (12.8%)	7 (14.0%)	10 (12.0%)	0.792
**Smoking status (*n* = 66)**				0.265
** Current/former**	17 (25.8%)	6 (18.8%)	11 (32.4%)	
** Never**	49 (74.2%)	26 (81.3%)	23 (67.6%)	
**ER status**				0.839
** Positive**	99 (74.4%)	38 (76.0%)	61 (73.5%)	
** Negative**	34 (25.6%)	12 (24.0%)	22 (26.5%)	
**PR status**				0.854
** Positive**	83 (62.4%)	32 (64.0%)	51 (61.4%)	
** Negative**	50 (37.6%)	18 (36.0%)	32 (38.6%)	
**Histological grade (*n* = 121)**				
** Grade 1**	2 (1.7%)	2 (4.3%)	0 (0%)	
** Grade 2**	47 (38.8%)	21 (44.7%)	26 (35.1%)	
** Grade 3**	72 (59.5%)	24 (51.1%)	48 (64.9%)	
**Pathological response (Miller-Payne classification) (*n* = 122)**				**<0.001**
** 1 (no change)**	2 (1.6%)	2 (4.2%)	0 (0%)	
** 2 (minor response)**	7 (5.7%)	5 (10.4%)	2 (2.7%)	
** 3 (partial response)**	28 (23.0%)	21 (43.8%)	7 (9.5%)	
** 4 (marked response)**	31 (25.4%)	16 (33.3%)	15 (20.3%)	
** 5 (complete response)**	54 (44.3%)	4 (8.3%)	50 (67.6%)	
**Pathological T stage**				0.150
** pT1**	46 (34.8%)	13 (26.5%)	33 (39.8%)	
** pT2**	74 (56.1%)	28 (57.1%)	46 (55.4%)	
** pT3**	8 (6.1%)	7 (14.3%)	1 (1.2%)	
** pT4**	4 (3.0%)	1 (2.0%)	3 (3.6%)	
**Pathological *N* stage**				**0.006**
** pN0**	45 (34.4%)	9 (18.4%)	36 (43.9%)	
** pN1**	65 (49.6%)	27 (55.1%)	38 (46.3%)	
** pN2**	20 (15.3%)	12 (24.5%)	8 (9.8%)	
** pN3**	1 (0.8%)	1 (2.0%)	0 (0%)	
**Surgical approach**				**0.002**
** Mastectomy**	118 (89.4%)	39 (78.0%)	79 (96.3%)	
** Lumpectomy**	14 (10.6%)	11 (22.0%)	3 (3.7%)	
**Neoadjuvant therapy**				
** Anthracycline-based**	80 (60.6%)	27 (54.0%)	53 (64.6%)	0.272
** Taxane-based**	128 (97.0%)	46 (92.0%)	83 (100.0%)	**0.019**
** Carboplatin-based**	15 (11.4%)	13 (26.0%)	2 (2.4%)	**<0.001**
**HER2-targeted therapy**				
** Trastuzumab alone**	47 (35.3%)	7 (14.0%)	40 (48.2%)	**<0.001**
** Trastuzumab + pertuzumab**	85 (63.9%)	42 (84.0%)	43 (51.8%)	**<0.001**

Significant *P* values are marked in bold.

Abbreviations: CAD, coronary artery disease; ECOG, Eastern Cooperative Oncology Group; ER, estrogen receptor; HER2, human epidermal growth factor receptor 2; PR, progesterone receptor; T-DM1, trastuzumab emtansine; pT, pathological tumor stage; pN, pathological nodal stage.

### Hematologic toxicity

Table 2 presents the incidence and severity of hematologic toxicities-including neutropenia, leukopenia, thrombocytopenia, and anemia-across both treatment groups. The incidence rates of neutropenia, leukopenia, and anemia showed no statistically significant intergroup differences. In contrast, any-grade thrombocytopenia occurred significantly more frequently with adjuvant T-DM1 and RT (35/50, 70.0%) compared to concurrent adjuvant trastuzumab and RT (30/83, 36.1%; *P* < .001). Furthermore, the severity distribution revealed significant intergroup differences (*P* = .035). Although grade 1 events predominated in both treatment groups, all higher-grade thrombocytopenia (grades 2-4) occurred exclusively in the T-DM1 arm. (grade 2: 14.3% vs 0%; grade 4: 5.7% vs 0%). Notably, among patients who developed thrombocytopenia, all events in the trastuzumab group were grade 1 (100.0%), whereas only 80.0% of T-DM1 cases were grade 1. Febrile neutropenia was not observed in either treatment arm.

**Table 2.** Hematologic toxicity by treatment group.

Toxicity	T-DM1 (*n* = 50)	Trastuzumab (*n* = 83)	*P* value
**Neutropenia, any grade**	19 (38.0%)	23 (27.7%)	0.250
** Grade 1**	10 (52.6%)	12 (52.2%)	0.953
** Grade 2**	6 (31.6%)	6 (26.1%)	—
** Grade 3**	1 (5.3%)	2 (8.7%)	—
** Grade 4**	2 (10.5%)	3 (13.0%)	—
**Leukopenia, any grade**	31 (62.0%)	57 (68.7%)	0.454
** Grade 1**	19 (61.3%)	39 (68.4%)	0.673
** Grade 2**	10 (32.3%)	13 (22.8%)	—
** Grade 3**	1 (3.2%)	4 (7.0%)	—
** Grade 4**	1 (3.2%)	1 (1.8%)	—
**Thrombocytopenia, any grade**	35 (70.0%)	30 (36.1%)	**<0.001**
** Grade 1**	28 (80.0%)	30 (100.0%)	**0.035**
** Grade 2**	5 (14.3%)	0 (0%)	—
** Grade 3**	0 (0%)	0 (0%)	—
** Grade 4**	2 (5.7%)	0 (0%)	—
**Anemia, any grade**	39 (78.0%)	66 (79.5%)	0.830
** Grade 1**	29 (74.4%)	50 (75.8%)	0.550
** Grade 2**	8 (20.5%)	15 (22.7%)	—
** Grade 3**	2 (5.1%)	1 (1.5%)	—
**Febrile neutropenia, any grade**	0 (0%)	0 (0%)	—

Significant *P* values are marked in bold.

Abbreviation: T-DM1, trastuzumab emtansine.

### Non-hematologic toxicity

Non-hematologic toxicity profiles are presented in Table 3. Notably, T-DM1 was associated with significantly higher rates of hepatic toxicity. ALT elevation occurred in 20.0% of patients receiving adjuvant T-DM1 and RT compared to 4.8% of those treated with concurrent adjuvant trastuzumab and RT (*P* = .008), whereas AST elevation was observed in 24.5% vs 2.4%, respectively (*P* < .001). All hepatic toxicities were mild to moderate (grade 1-2), with no severe hepatotoxicity (grade 3-4) documented in either treatment group. Neuropathy rates were comparable between groups (4.0% vs 4.8%, *P* = 1.000), as were dermatologic toxicities, with skin reactions affecting approximately one-third of patients in both arms (32.7% vs 33.8%, *P* = 1.000). Pulmonary toxicity patterns were found to differ by timing. Early pulmonary toxicity was significantly more frequent in the T-DM1 group (48.8% vs 24.1%, *P* = .017), though most cases were grade 1 severity (75.0% vs 84.6% of affected patients), with only 1 grade 3 event occurring in the T-DM1 arm. Conversely, late pulmonary toxicity exhibited a non-significant trend toward higher incidence in the trastuzumab group (52.7% vs 35.0%, *P* = .099).

**Table 3.** Non-hematologic toxicity by treatment group.

Toxicity	T-DM1 (*n* = 50)	Trastuzumab (*n* = 83)	*P* value
**Hepatic toxicity**			
** ALT elevation, any grade**	10 (20.0%)	4 (4.8%)	**0.008**
** Grade 1**	8 (80.0%)	3 (75.0%)	1.000
** Grade 2**	2 (20.0%)	1 (25.0%)	—
** AST elevation, any grade**	12 (24.5%)	2 (2.4%)	**<0.001**
** Grade 1**	11 (91.7%)	2 (100.0%)	1.000
** Grade 2**	1 (8.3%)	0 (0%)	—
**Neurologic toxicity**			
** Neuropathy, any grade**	2 (4.0%)	4 (4.8%)	1.000
**Dermatologic toxicity**			
** Skin toxicity, any grade**	16 (32.7%)	27 (33.8%)	1.000
** Grade 1**	12 (75.0%)	23 (85.2%)	0.680
** Grade 2**	4 (25.0%)	4 (14.8%)	—
**Pulmonary toxicity**			
** Early pulmonary toxicity (0-6 months), any grade**	20/41 (48.8%)	13/54 (24.1%)	**0.017**
** Grade 1**	15 (75.0%)	11 (84.6%)	0.680
** Grade 2**	4 (20.0%)	2 (15.4%)	—
** Grade 3**	1 (5.0%)	0 (0%)	—
** Late pulmonary toxicity (6-12 months), any grade**	14/40 (35.0%)	29/55 (52.7%)	0.099
** Grade 1**	14 (100.0%)	28 (96.6%)	1.000
** Grade 4**	0 (0%)	1 (3.4%)	—

Significant *P* values are marked in bold.

Abbreviations: ALT, alanine aminotransferase; AST, aspartate aminotransferase; T-DM1, trastuzumab emtansine.

### Associations of adjuvant treatment types with treatment-related toxicities

In univariable analysis, adjuvant treatment type was identified as a significant predictor for several toxicities. Specifically, relative to trastuzumab, T-DM1 was associated with higher odds of thrombocytopenia (OR, 4.12; 95% CI, 1.94-8.74; *P* < .001), ALT elevation (OR, 4.94; 95% CI, 1.46-16.74; *P* = .008), AST elevation (OR, 13.14; 95% CI, 2.80-61.68; *P* < .001), and early pulmonary toxicity (OR, 3.00; 95% CI, 1.25-7.19; *P* = .012) (Figure 2). To assess independence, we performed multivariable logistic regression analyses after adjustment for variables showing a *P*-value <.10 in the univariable analysis. Notably, treatment with T-DM1 was not retained as an independent predictor in the adjusted model; however, a poorer ECOG performance status (ECOG 1 vs 0) emerged as the sole independent predictor of early pulmonary toxicity (adjusted OR, 11.9; 95% CI, 1.06-125.0; *P* = .044). In sensitivity analyses adjusting for pertuzumab exposure, associations between T-DM1 and key toxicities remained significant. Specifically, thrombocytopenia (adjusted OR, 3.85; 95% CI, 1.76-8.42; *P* = .001), ALT elevation (adjusted OR, 4.12; 95% CI, 1.18-14.35; *P* = .026), and early pulmonary toxicity (adjusted OR, 2.64; 95% CI, 1.08-6.47; *P* = .034). In the subset analysis restricted to patients receiving dual HER2 blockade (T-DM1, *n* = 42; trastuzumab + pertuzumab, *n* = 43), similar toxicity patterns were observed, as follows: thrombocytopenia (69.0% vs 37.2%, *P* = .004) and early pulmonary toxicity (50.0% vs 23.3%, *P* = .012) remained significantly higher in the T-DM1 group.

**Figure 2.**
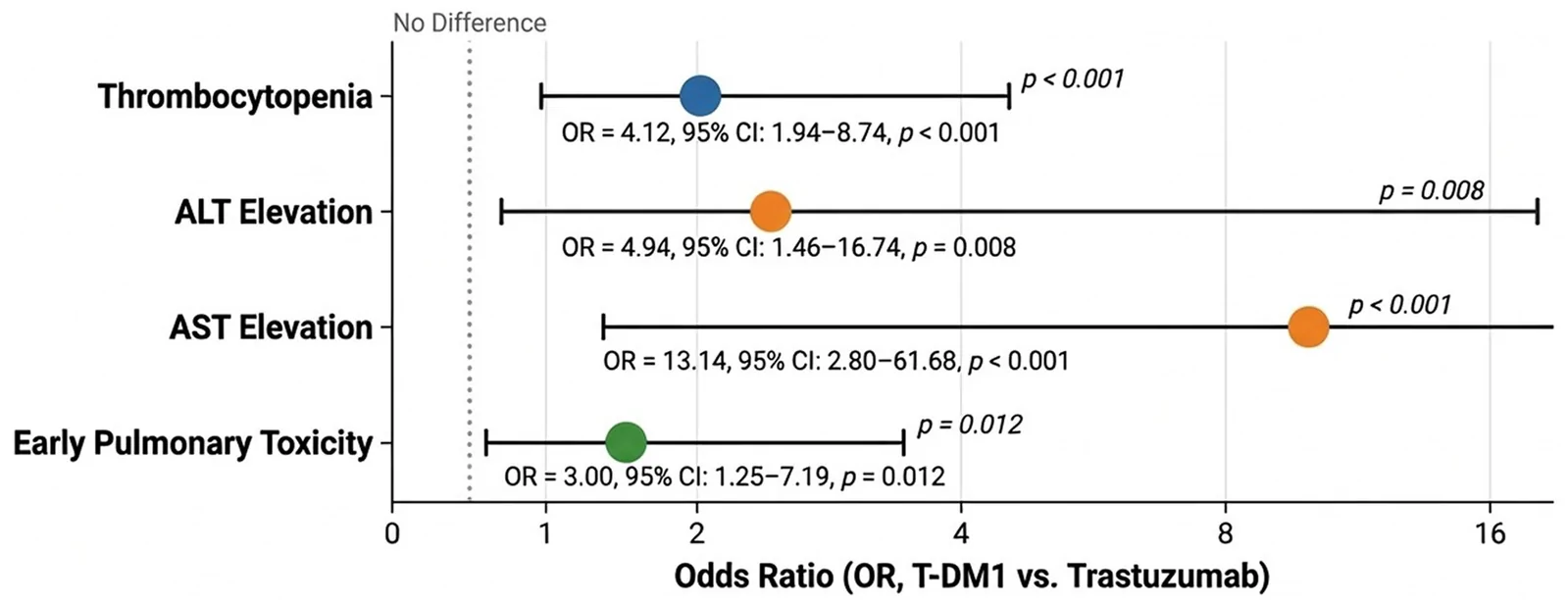
Forest plot of odds ratios for key toxicities comparing the T-DM1 vs trastuzumab groups. ALT, alanine aminotransferase; AST, aspartate aminotransferase; CI, confidence interval; OR, odds ratio; T-DM1, trastuzumab emtansine.

### Clinical management

Most adverse events were effectively managed with standard supportive care without requiring permanent treatment discontinuation. Thrombocytopenia was addressed through platelet transfusions when clinically indicated, and elevated hepatic enzymes resolved following dose modifications or temporary treatment interruptions. No treatment-related deaths occurred in either study arm during the follow-up period.

## Discussion

To our knowledge, this real-world, retrospective, multicenter analysis represents the largest study to date evaluating the safety of concurrent adjuvant T-DM1 and RT in patients with HER2-positive early-stage BC. Our findings demonstrate that this combination is clinically feasible, though it exhibits a distinct safety profile characterized by significantly higher rates of hematologic, hepatic, and early pulmonary toxicities compared to standard adjuvant trastuzumab with RT. Collectively, our data provide essential safety information as adjuvant T-DM1 becomes increasingly adopted in routine clinical practice^17^ following the KATHERINE trial,^7^ which established adjuvant T-DM1 as standard care for patients with residual disease after neoadjuvant therapy. Recent advances have introduced trastuzumab deruxtecan (T-DXd) as a potentially superior alternative to T-DM1 in high-risk HER2-positive BC.^22^ However, T-DM1 remains widely used and continues to be the standard option in several regions, making real-world safety data with concurrent RT critically important.

Our findings build upon limited prior literature evaluating T-DM1 with concurrent RT. Zolcsák et al.^15^ reported preliminary data from 14 patients with nonmetastatic HER2-positive BC showing that concurrent T-DM1 and RT was generally well tolerated, with mostly mild radiodermatitis and acceptable cardiac and hepatic toxicity profiles. Salvestrini et al.^16^ conducted a systematic review and meta-analysis of 9 studies showing that combining T-DM1 with radiotherapy in BC has an overall acceptable safety profile, with low rates of severe pneumonitis (< 1%) and skin toxicity but a noteworthy 17% incidence of grade 3 or higher radionecrosis. Our observed early pulmonary toxicity rate of 48.8% is higher than previously reported, likely reflecting differing endpoint definitions. Accordingly, we captured all radiographic abnormalities, whereas most prior studies reported only clinically overt pneumonitis. Notably, the meta-analysis did not include direct comparisons with trastuzumab-based regimens,^16^ a gap that our study specifically addresses. Few studies have directly compared T-DM1 vs trastuzumab toxicity in similar populations. In the KATHERINE trial,^7^ T-DM1 was associated with higher overall thrombocytopenia rates (29% vs 2.4% with trastuzumab), similar to our findings, though our concurrent RT population showed markedly higher rates (70%). Our findings extend these observations by demonstrating that when combined with concurrent RT, toxicity differences between T-DM1 and trastuzumab may become more pronounced, particularly for hematologic and pulmonary outcomes.

Our findings are consistent with, and further build on, previous reports of T-DM1 toxicity.^13^^,^^23^

The most clinically significant finding from the current study was the markedly elevated thrombocytopenia incidence in the T-DM1 cohort (70.0%), which substantially exceeds the 28-32% rates typically reported with T-DM1 in non-irradiated populations^13^^,^^24^—likely due to concurrent RT. Hepatic enzyme elevations (ALT, 20%; AST, 24.5%) in our study fell within the upper limits of the established 15%-25% range,^13^ suggesting potential additive effects. The observed toxicity patterns reflect plausible biological mechanisms underlying drug-radiation interactions. For thrombocytopenia, the maytansinoid payload DM1 inhibits microtubule polymerization,^25^ a process essential for megakaryocyte maturation and platelet production.^26^ Concurrent RT likely exacerbates this effect through multiple pathway-including radiation-induced production of proinflammatory cytokines that suppress megakaryopoiesis; potential bone marrow exposure within radiation fields, particularly with regional nodal irradiation.^27^ These synergistic mechanisms may explain the higher thrombocytopenia rates observed compared to historical T-DM1 monotherapy data. However, the severity distribution of thrombocytopenic events provides reassurance for clinical management. Although all higher-grade thrombocytopenia events (grades 2-4) occurred exclusively in the T-DM1 group, most cases were limited to grade 1 (80.0% in the T-DM1 group). Nonetheless, the occurrence of grade 4 thrombocytopenia in 5.7% of T-DM1 patients underscores the need for proactive monitoring protocols.

The significantly increased hepatic enzyme elevations represent an anticipated safety signal^28^ that reflects additive hepatotoxic effects when T-DM1 is combined with concurrent RT, as both modalities can independently cause liver enzyme elevations. Both T-DM1 and RT independently may affect hepatic function through distinct mechanisms. DM1 undergoes extensive hepatic metabolism via CYP3A4, and elevated systemic drug exposure can precipitate hepatocyte injury.^28^ Conversely, concurrent RT—even when liver is not directly in treatment field-can induce systemic inflammatory responses with elevated circulating cytokines that modulate hepatic drug-metabolizing enzyme expression and activity.^29^^,^^30^ While all observed hepatic toxicities were grade 1-2 and reversible, the potential for progression necessitates careful surveillance-including baseline liver function tests before treatment initiation and ALT/AST monitoring before each T-DM1 cycle during concurrent phase.

The 48.8% incidence of early pulmonary toxicity in the T-DM1 group—though predominantly grade 1—represents a clinically significant finding with potential implications for patient monitoring and counseling.^31^ For pulmonary toxicity, potential mechanisms include direct radiosensitization of pneumocytes and pulmonary endothelium by T-DM1, enhancing radiation-induced cellular injury; microtubule inhibition by DM1 affecting type II pneumocyte surfactant production and repair capacity; enhanced inflammatory cascade activation, with radiation-induced lung injury amplified by T-DM1-associated cytokine release; and potential impairment of normal tissue repair mechanisms.^32^^,^^33^ In our study, the intriguing trend toward higher late pulmonary toxicity in the trastuzumab group (52.7% vs 35.0%, *P* = .099) suggests distinct pathophysiologic mechanisms underlying early vs late radiation-induced lung injury. This temporal shift may reflect T-DM1’s predominant impact on acute inflammatory processes^34^ rather than the fibrotic mechanisms underlying late pulmonary complications. From a practical perspective, during the initial phase (0-6 months), systematic clinical evaluation for dyspnea, cough, and functional decline should be conducted, with a low threshold for chest CT imaging in symptomatic individuals and consideration of pulmonary function testing in patients with clinically significant symptoms. In the later phase (beyond 6 months), ongoing surveillance should continue regardless of treatment assignment, including CT imaging at 12-24 months. Notably, our multivariable analysis identified poorer ECOG performance status as the sole independent predictor of early pulmonary toxicity, suggesting that patient fitness significantly influences tolerance to concurrent adjuvant T-DM1 and RT.

Clinical decision-making requires weighing toxicity risks against efficacy benefits. The predominantly mild-to-moderate hematologic, hepatic, and pulmonary toxicities observed in our study—which appeared manageable with supportive care without treatment discontinuation—represent acceptable tradeoffs given the expected survival advantages.^7^ However, patient selection remains critical. For patients with excellent performance status (ECOG 0), normal organ function, and adequate bone marrow reserve, concurrent T-DM1 and RT can improve systemic and local control while minimizing treatment duration. Conversely, for patients with compromised performance status (ECOG ≥1), baseline cytopenias, hepatic dysfunction, or preexisting pulmonary disease, sequential administration may be preferable to reduce toxicity burden. In this scenario, shared decision-making incorporating individual risk factors, treatment goals, and patient preferences should guide management.

Several limitations inherent in our study warrant consideration, primarily stemming from the retrospective, non-randomized design. Accordingly, selection bias is evident in the T-DM1 cohort’s higher-risk features and treatment intensity. Despite multivariable adjustment, residual confounding may overestimate toxicity, rendering our findings hypothesis-generating. However, the baseline imbalances observed between treatment groups reflect real-world treatment patterns where T-DM1 is reserved for patients with residual disease after neoadjuvant therapy. We also acknowledge that poorer ECOG performance status may reflect subclinical organ dysfunction or reduced physiologic reserve, potentially increasing susceptibility to treatment-related adverse events. Higher nodal disease burden typically necessitates more extensive RT fields with larger treatment volumes, which may increase normal tissue exposure and radiation-related toxicity. The observed baseline differences in the 2 study groups limit our ability to definitively attribute toxicity differences solely to the T-DM1 vs trastuzumab comparison rather than to underlying patient and disease characteristics. Generalizability is further constrained by the single-region setting and insufficient statistical power to exclude type II error for rare adverse events. Moreover, the 1-year follow-up can capture acute toxicity but precludes assessment of late-onset complications. Methodologically, our pulmonary safety data were limited by the absence of blinded, independent radiologic review and standardized metrics, complicating the distinction between treatment-induced changes and pneumonitis. Finally, the heterogeneous retrospective follow-up necessitated the use of binary rather than time-to-event endpoints.

## Conclusion

The concurrent administration of adjuvant T-DM1 and RT was clinically feasible in HER2-positive early-stage BC. Although the toxicity profile appears manageable, specific safety signals—notably thrombocytopenia, hepatotoxicity, and pulmonary complications—necessitate vigilant surveillance. Our findings provide a preliminary framework for clinical decision-making; however, prospective validation is essential to definitively characterize safety and refine monitoring protocols.

## Data Availability

All data that support the findings of this study are accessible from the corresponding author upon reasonable request.

## References

[1] Bartsch R , Cameron D, Ciruelos E, et al Expert recommendations on treatment sequencing and challenging clinical scenarios in human epidermal growth factor receptor 2-positive (HER2-positive) metastatic breast cancer. Cancer Treat Rev. 2025;132:102853. 10.1016/j.ctrv.2024.102853 39580869

[2] Giffoni de Mello Morais Mata D , Chehade R, Hannouf MB, et al Appraisal of systemic treatment strategies in early HER2-positive breast cancer—a literature review. Cancers (Basel). 2023;15:4336. 10.3390/cancers15174336 PMC10486709 37686612

[3] Li X , Zhang X, Yin S, Nie J. Challenges and prospects in HER2-positive breast cancer-targeted therapy. Crit Rev Oncol Hematol. 2025;207:104624. 10.1016/j.critrevonc.2025.104624 39826885

[4] Swain SM , Shastry M, Hamilton E. Targeting HER2-positive breast cancer: advances and future directions. Nat Rev Drug Discov. 2023;22:101-126. 10.1038/s41573-022-00579-0 PMC9640784 36344672

[5] Hendrix N , Oestreicher N, Lalla D, et al Residual disease burden after neoadjuvant therapy among US patients with high-risk HER2-positive early-stage breast cancer: a population effectiveness model. Clin Breast Cancer. 2022;22:781-791. 10.1016/j.clbc.2022.08.012 36220724

[6] Fernandez-Martinez A , Tanioka M, Ahn SG, et al Prognostic value of residual disease (RD) biology and gene expression changes during the neoadjuvant treatment in patients with HER2-positive early breast cancer (EBC). Ann Oncol. 2025;36:403-413. 10.1016/j.annonc.2024.12.010 PMC11949722 39706338

[7] von Minckwitz G , Huang C-S, Mano MS, et al; KATHERINE Investigators. Trastuzumab emtansine for residual invasive HER2-positive breast cancer. N Engl J Med. 2019;380:617-628. 10.1056/NEJMoa1814017 30516102

[8] Yeh Y , Chen C, Ko Y. Effectiveness and cost-effectiveness of trastuzumab emtansine in women with HER2-positive locally advanced or metastatic breast cancer: a systematic review and meta-analysis. J Cancer Res Ther. 2022;18:1061-1072. 10.4103/jcrt.jcrt_1095_21 36149162

[9] Dowling GP , Keelan S, Toomey S, Daly GR, Hennessy BT, Hill ADK. Review of the status of neoadjuvant therapy in HER2-positive breast cancer. Front Oncol. 2023;13:1066007. 10.3389/fonc.2023.1066007 PMC9923093 36793602

[10] Kolářová I , Melichar B, Sirák I, et al The role of adjuvant radiotherapy in the treatment of breast cancer. Curr Oncol. 2024;31:1207-1220. 10.3390/curroncol31030090 PMC10969207 38534923

[11] Debbi K , Grellier N, Loganadane G, et al Interaction between radiation therapy and targeted therapies in HER2-positive breast cancer: literature review, levels of evidence for safety and recommendations for optimal treatment sequence. Cancers (Basel). 2023;15:2278. 10.3390/cancers15082278 PMC10137001 37190205

[12] Belkacémi Y , Gligorov J, Ozsahin M, et al Concurrent trastuzumab with adjuvant radiotherapy in HER2-positive breast cancer patients: acute toxicity analyses from the french multicentric study. Ann Oncol. 2008;19:1110-1116. 10.1093/annonc/mdn029 18344537

[13] Kowalczyk L , Bartsch R, Singer CF, Farr A. Adverse events of trastuzumab emtansine (T-DM1) in the treatment of HER2-positive breast cancer patients. Breast Care (Basel). 2017;12:401-408. 10.1159/000480492 PMC5803744 29456473

[14] Wan G , Yang L, Wang Q, Xu G. T-DM1 with concurrent radiotherapy in HER2-positive breast cancer: preclinical evaluation and mechanisms, prediction, and exploration of adverse effects. Discov Oncol. 2025;16:857. 10.1007/s12672-025-02239-2 PMC12098256 40402389

[15] Zolcsák Z , Loirat D, Fourquet A, Kirova YM. Adjuvant trastuzumab emtansine (T-DM1) and concurrent radiotherapy for residual invasive HER2-positive breast cancer: single-center preliminary results. Am J Clin Oncol. 2020;43:895-901. 10.1097/COC.0000000000000769 33027084

[16] Salvestrini V , Kim K, Caini S, et al Safety profile of trastuzumab-emtansine (T-DM1) with concurrent radiation therapy: a systematic review and meta-analysis. Radiother Oncol. 2023;186:109805. 10.1016/j.radonc.2023.109805 37437610

[17] Krasniqi E , Filomeno L, Arcuri T, et al Tolerability and preliminary outcomes of adjuvant T-DM1 in HER2-positive breast cancer after neoadjuvant therapy: the ATD study. Cancers (Basel). 2024;16:4104. 10.3390/cancers16234104 PMC11639934 39682290

[18] Ogston KN , Miller ID, Payne S, et al A new histological grading system to assess response of breast cancers to primary chemotherapy: prognostic significance and survival. Breast. 2003;12:320-327. 10.1016/S0960-9776(03)00106-1 14659147

[19] Oken MM , Creech RH, Tormey DC, et al Toxicity and response criteria of the Eastern cooperative oncology group. Am J Clin Oncol. 1982;5:649-656. 7165009

[20] Freites-Martinez A , Santana N, Arias-Santiago S, Viera A. Using the common terminology criteria for adverse events (CTCAE—version 5.0) to evaluate the severity of adverse events of anticancer therapies. Actas Dermosifiliogr (Engl Ed). 2021;112:90-92. 10.1016/j.ad.2019.05.009 32891586

[21] Geyer CE Jr , Park YH, Shao Z, et al Trastuzumab deruxtecan (T-DXd) vs trastuzumab emtansine (T-DM1) in patients with high-risk human epidermal growth factor receptor 2-positive primary breast cancer with residual invasive disease after neoadjuvant therapy: Interim analysis of DESTINY-Breast05. Presented at ESMO Congress 2025; October 18, 2025. Presidential Symposium I, LBA1.

[22] Harbeck N , Modi S, Pusztai L, et al; DESTINY-Breast11 Trial Investigators Neoadjuvant trastuzumab deruxtecan alone or followed by paclitaxel, trastuzumab, and pertuzumab for high-risk HER2-positive early breast cancer (DESTINY-Breast11): a randomised, open-label, multicentre, phase 3 trial. Ann Oncol. 2026;37:166-179. 10.1016/j.annonc.2025.10.019 41130363

[23] Verma S , Miles D, Gianni L, et al; EMILIA Study Group. Trastuzumab emtansine for HER2-positive advanced breast cancer. N Engl J Med. 2012;367:1783-1791. 10.1056/NEJMoa1209124 PMC5125250 23020162

[24] Zhang J , Yang Y, Chen R, et al Assessment of racial differences in the incidence of thrombocytopenia induced by trastuzumab emtansine: a systematic review and meta-analysis. Ann Transl Med. 2021;9:1139. 10.21037/atm-21-2763 PMC8350666 34430580

[25] Lopus M , Oroudjev E, Wilson L, et al Maytansine and cellular metabolites of antibody-maytansinoid conjugates strongly suppress microtubule dynamics by binding to microtubules. Mol Cancer Ther. 2010;9:2689-2699. 10.1158/1535-7163.MCT-10-0644 PMC2954514 20937594

[26] Thon JN , Devine MT, Jurak Begonja A, Tibbitts J, Italiano JE Jr. High-content live-cell imaging assay used to establish mechanism of trastuzumab emtansine (T-DM1)–mediated inhibition of platelet production. Blood. 2012;120:1975-1984. 10.1182/blood-2012-04-420968 PMC3437592 22665936

[27] Takeda K , Umezawa R, Yamamoto T, et al Acute hematologic toxicity of radiation therapy: a comprehensive analysis and predictive nomogram. J Radiat Res. 2023;64:954-961. 10.1093/jrr/rrad069 PMC10665302 37740569

[28] Cobert AM , Helms C, Larck C, Moore DC. Risk of hepatotoxicity with trastuzumab emtansine in breast cancer patients: a systematic review and meta-analysis. Ther Adv Drug Saf. 2020;11:2042098620915058. 10.1177/2042098620915058 PMC7175051 32341779

[29] Schaue D , Kachikwu EL, McBride WH. Cytokines in radiobiological responses: a review. Radiat Res. 2012;178:505-523. 10.1667/RR3031.1 PMC3723384 23106210

[30] Wang X , Rao J, Tan Z, Xun T, Zhao J, Yang X. Inflammatory signaling on cytochrome P450-mediated drug metabolism in hepatocytes. Front Pharmacol. 2022;13:1043836. 10.3389/fphar.2022.1043836 PMC9637984 36353494

[31] Egloff H , Kidwell KM, Schott A. Ado-trastuzumab emtansine–induced pulmonary toxicity: a single-institution retrospective review. Case Rep Oncol. 2018;11:527-533. 10.1159/000491574 PMC6120401 30186135

[32] Endo Y , Mohan N, Dokmanovic M, Wu WJ. Mechanisms contributing to ado-trastuzumab emtansine-induced toxicities: a gateway to better understanding of ADC-associated toxicities. Antib Ther. 2021;4:55-59. 10.1093/abt/tbab005 PMC7998064 33937626

[33] Salvestrini V , Kim K, Caini S, et al Safety profile of trastuzumab-emtansine (T-DM1) with concurrent radiation therapy: a systematic review and meta-analysis. Radiother Oncol. 2023;186:109805. 10.1016/j.radonc.2023.109805 37437610

[34] Wan G , Yang L, Wang Q, Xu G. T-DM1 with concurrent radiotherapy in HER2-positive breast cancer: preclinical evaluation and mechanisms, prediction, and exploration of adverse effects. Discov Oncol. 2025;16:857. 10.1007/s12672-025-02239-2 PMC12098256 40402389

